# Low-cost test rig for characterization of photocatalytic planar materials using photonically sized UV-A LED light sources

**DOI:** 10.1016/j.ohx.2023.e00487

**Published:** 2023-11-04

**Authors:** Tobias Schnabel, Robert Honke, Andreas Schmid, Simon Mehling, Rene' Göhring, Oldrich Simek, Axel Wolfram, Andre Wetterauer, Christian Springer

**Affiliations:** a Hof University of Applied Science, Alfons-Goppel Platz 1, 95028, Hof; b Erfurt University of Applied Science, Altonaer Straße 25, 99085, Erfurt

**Keywords:** UV-LED, Photocatalytic Material, UV–VIS spectroscopy, AOP-Process, Titanium Dioxide, Photonics

## Abstract

In the presented studies, a system for the characterization of planar photocatalysts was developed and tested. In the system, reference substances can be studied online with regard to their degradability and adsorption on photocatalytic materials. In order to perform accurate calculations of the quantum and photon efficiency of the catalysts, the LED arrays used were adjusted in their spacing by simulations so that a homogeneous light field is imaged on the catalysts. The system was tested with respect to measurement accuracy and reproducibility and the photocatalytic degradation of methylene blue, methyl orange and rhodamine B was investigated. Exemplarily, the reaction kinetics, photolysis and adsorption on the tested photocatalysts were determined for these compounds and the calculation was presented in detail. The exact construction plans and circuits as well as the sensors and their programming are presented in detail and should encourage other scientists to replicate the experimental setup, since especially in the field of photocatalysis research, often the results of publications cannot be compared with each other.

## Graphical abstract

**Figure:**
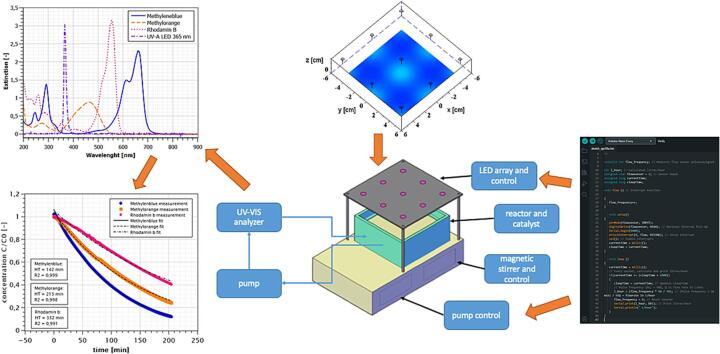

**Specifications table**.

**Table:** 

Hardware name	test rig for characterization of photocatalytic degradation reactions and materials
Subject area	• Engineering and materials science • Chemistry and biochemistry • Environmental chemistry
Hardware type	• Measuring physical properties and in-lab sensors • Mechanical engineering and materials science • Investigation of catalytic properties
Closest commercial analog	*No commercial analog is available.*
Open source license	*Creative Commons Attribution 4.0 International*
Cost of hardware	*840 €.*
Source file repository	*https://doi.org/10.5281/zenodo.8066013*

## 1 Hardware in context

The photocatalytic effect on semiconductors, often titanium dioxide, and the production of efficient catalyst coatings has been the subject of countless studies since the discovery of photocatalytic water splitting by.

[1] Recently, photocatalysis is gaining greater importance in science due to energy scarcity as well as the possibility of solar hydrogen generation [2], [3]. In addition to energy applications, photocatalysts can be used for oxidative purification of organic pollutants in indoor air, degradation of nitrogen oxides in urban air, and purification of wastewater and water [4], [5], [6], [7]. A major problem in many studies of photocatalytic materials is the use of ill-defined light sources and experimental setups in the characterization of planar catalyst layers and materials [8], [9]. The data can often not be compared in a qualified manner since polychromatic light sources such as mercury low pressure lamps or xenon short arc lamps are used for the investigation. The same applies to the measurement of photocurrents via the three-electrode potentiostat method [3]. Following this necessity, various standardized test methods have been developed (including the use of the dye methylene blue (DIN 52980:2021–10, ISO 10678:2010). Here, a cylindrical reactor cell is applied to a coated test sample, mixed periodically (every 20 min) and the absorbance curve of the methylene blue solution is determined online or periodically. Specifications for conditioning the specimens prior to testing and blind tests are also included here. However, specifications for the arrangement of the light sources are missing here. Although the radiation to be supplied (10 W/m^2^) is defined and its punctual measurement is prescribed, the distribution of the light field is not taken into account here. Furthermore, activation-limited kinetics is assumed and represented by a linear analysis of the reaction rate, which is only correct for low activity catalyst materials [10].

In order for more scientists to have access to test systems that provide comparable and photonically calculable values in the future, this manuscript presents the setup of a test rig to determine the photocatalytic activity of planar materials. The light sources were designed in such a way that a well calculable, homogeneous light field can be generated. The calculation is provided with this work as a finished programming, so that defined luminous fields can be generated even without radiation measurement technology. The LED array can be controlled in its intensity by a PWM regulation of the light source by means of microcontrollers and can even be used in pulsed mode. Furthermore, a low-cost periphery with sample pump, flow measurement and magnetic stirrer with a device for holding the catalyst samples is presented. Simple automation interfaces are provided for all components, enabling efficient measurement operation with high data density. Due to the modular design, the setup is easily adaptable to the respective investigation questions and measurement methods. All components can be easily replicated with little financial means using Arduino Uno microcontrollers.

## 2 Hardware description

The presented test stand consists of two parts. One part is a controllable and radiometrically measured and simulated UV-A light source consisting of 9 high power UV-A LEDs with a peak wavelength of 365 nm. The LEDs are driven by a PWM controllable constant current source with Arduino microcontroller, which allows adjustment of the photon flux and also pulsing of the LEDs. Furthermore, the arrangement of the LEDs provides an almost homogeneous light field, so that additional optical components (especially concentrating lenses) are not necessary. The second part of the test rig consists of a Plexiglas reaction vessel with a volume of 500 ml and a Plexiglas frame for mounting the catalyst samples. The reactor was sealed using acrifix glue for the Plexiglas parts and PTFE tape on the inlet and outlet. The catalyst samples were installed at a distance of 5 mm below the water surface to ensure sufficient perfusion of the catalyst. This reaction vessel is located below the light source and can be mixed with a magnetic stirrer. The catalyst sample (here titanium dioxide immobilized on a glass fiber grid) covers the entire reactor surface. It has a mesh size of 0.5 mm and a fiber thickness of 1 mm. Appropriate openings, as described in DIN 52980:2021–10, must be provided for non– permeable catalyst shapes. By providing an almost homogeneous light field over a wide area, it is possible to easily adapt the reactor shape depending on the specific research question. The prerequisite for this is that a sufficient distance to the LED array is maintained. Next to the magnetic stirrer is a gear pump that is connected to the vessel and continuously pumps sample solution through a quartz flow cell that can be used in any laboratory UV–VIS spectrometer for dynamic time-resolved measurement of dye concentration. The pump is optional, as manual sampling from the reaction volume and off-line measurement is also possible. This is particularly recommended for the investigation of (nano-)particulate catalysts, which can be damaged by the gear pump and make online UV–VIS measurement difficult. However, the possibility of automated online measurement offers the possibility of a higher temporal resolution without affecting the reaction volume. Thus, very precise degradation, photolysis and adsorption curves can be determined. When using simultaneous measuring CCD UV–VIS spectrometers, even complete spectra can be measured in high temporal resolution, which can give indications on possible transformation products and allows subsequent calculations and evaluations over several wavelengths. This would also allow turbidity compensation for (nano)-particulate catalysts. Altogether, the setup presented offers its users the following advantages, in addition to financial savings:

- Photocatalytic experiments with defined homogeneous light fields for improved comparability of experimental data.
- Easy integration of online measurement systems for improved temporal recording of reaction kinetics.
- Reactor design with programmable components for automated experimental procedures.

## 3 Design files

### 3.1 Electronics

**Fig. 1:**
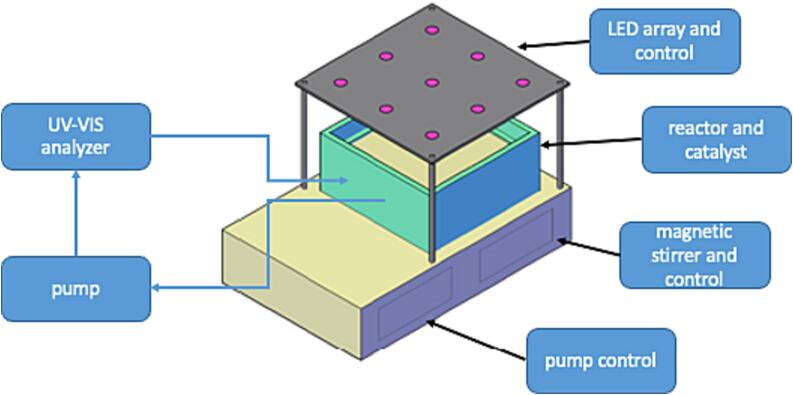
Schematic of the experimental Set-Up.

Fig. 1 shows the entire system including the UV–VIS spectrometer. The basic housing contains two Joy-IT 50 V/15A JT-DPS5015 power supply modules, which are supplied with 12 V voltage at a maximum current consumption of 2A via an external laboratory power supply. Alternatively, a fixed DC power supply can also be integrated into the housing. The housing is a waterproof housing with the dimensions (W XH X D). A magnetic stirrer consisting of a 12 V computer fan and two neodymium magnets is built into the right side of the housing (any 8 cm computer fan can be used). It is operated at 4 V voltage in the experiments and supplied via the right-hand power supply module. On the left side there is the pump for feeding the measuring solution through the UV–VIS Spectrometer. A 12 V gear pump (Extron 2141873) is used here, which is operated at 4 V by the left power supply module. This pump is connected to a flow sensor (VF-S401) via silicone tubing (5 mm ID). The flow sensor is read out via an Arduino Uno. In parallel to the power supply modules a step-down converter (LM 2696) is connected to supply the Arduino Uno with voltage. This can be omitted if the Arduino is only read out via the PC. If a display is used, the Arduino and the display can also be operated via the step-down converter without USB connection. The flow of the pump can be adjusted via the flow meter and the power supply. The connections of the pump are led out of the housing via a 3 mm ID plexiglass tube, so that the flow cell (Hellma Analytics 176.700 QS, 5 mm) can be connected. The circuit diagram for this part is shown in the design files.

**Fig. 2:**
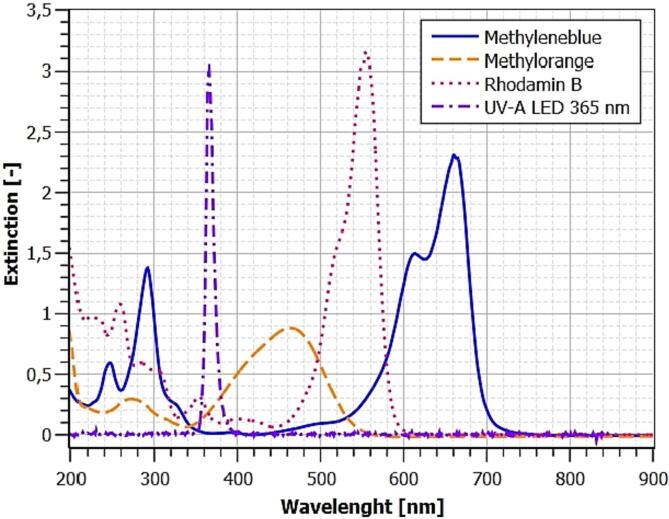
UV and VIS spectra of dyes and LEDs used.

**Fig. 3:**
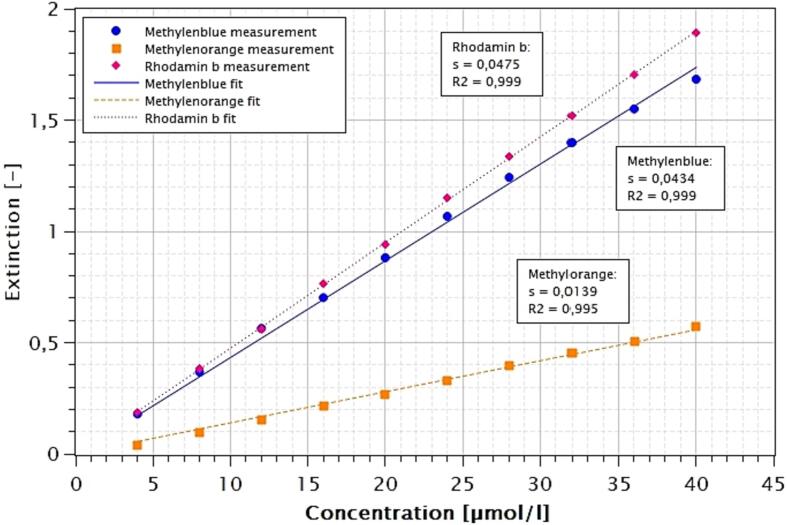
Calibration of photometric measurements (s = slope linear regression without intercept; R2 = coefficient of determination).

Fig. 3 shows the circuit diagram of the LED light source. 9 Seoul CUN66A1B LEDs with 2 W electrical and 1 W radiometric power at 365 nm wavelength were used. The 9 LEDs are arranged in a 50 mm grid, each in three rows of three LEDs squared and centered on a 200 × 200 mm aluminum plate with a thickness of 2 mm using thermal adhesive. The LEDs are connected in series and connected to a Meanwell 500 mA constant current source with PWM dimming input. The dimming input of the constant current source is connected to a PWM output of an Arduino Uno, which in turn can be supplied with voltage via a step-down converter. All loads (input constant current source and input step-down converter) are supplied via 4 mm sockets with a DC voltage of 36 V by means of an external laboratory power supply. Using the PWM input of the constant current source, pulse and current programs for LED operation can be retrieved via the Arduino Uno.

### 3.2 Program code

The given Arduino sketches represent the minimal functions of the two Arduino Uno used. Sketch 1 is the PWM control of the UV-A LEDs and allows via the PWM signals an adjustment of the constant current of the light source, where 255 corresponds to the maximum current of 500 mA and can be decreased linearly. A simple extension of the code allows the LED array to be pulsed, or timed LED programs can be implemented. Sketch 2 is for operating the flow sensor and reading out the values via the Arduino Serial Monitor.

### 3.3 3D printing and CAD files

The CAD files and 3D print files show on the one hand the Plexiglas parts for the reaction cell and on the other hand the measuring template, which can be used to measure the UV light field. All files are listed in the table below.

### 3.4 Design files summary

**Table:** 

**Design file name**	**File type**	**Open source licence**	**Location of the file**
*01_reactor_construction_acrylglas*	*Figure*	*Creative Commons Attribution 4.0 International*	*https://doi.org/10.5281/zenodo.8066013*
*02_electronics_pump_stirrer*	*Figure*	*Creative Commons Attribution 4.0 International*	*https://doi.org/10.5281/zenodo.8066013*
*03_electronic_UV_array*	*Figure*	*Creative Commons Attribution 4.0 International*	*https://doi.org/10.5281/zenodo.8066013*
*04_code_flow_sensor*	*Code*	*Creative Commons Attribution 4.0 International*	*https://doi.org/10.5281/zenodo.8066013*
*05_code_PWM_LED*	*Code*	*Creative Commons Attribution 4.0 International*	*https://doi.org/10.5281/zenodo.8066013*
*06_code_SCILAB_LED_SIM*	*Code*	*Creative Commons Attribution 4.0 International*	*https://doi.org/10.5281/zenodo.8066013*

## 4 Bill of materials summary

**Table:** 

**Component**	**Number**	**Cost per unit -currency**	**Total cost -currency**	**Source of materials**	**Material type**
*Arduino Uno*	2	25 €	50 €	Amazon	Electronics
Joy-IT 50 V/15A JT-DPS5015	2	55 €	110 €	Amazon	Electronics
12 V computer fan	1	10 €	10 €	Amazon	Electronics
neodymium magnets	2	1 €	2 €	Amazon	Electronics
Extron 2,141,873	1	33 €	33	Extron	Electronics
flow sensor VF-S401	1	12 €	12 €	Amazon	Electronics
step-down converter LM2696	1	9 €	9 €	Amazon	Electronics
Hellma Analytics 176.700 QS, 5 mm	1	571 €	571 €	Analytics Shop	Laboratory equipment
Seoul CUN66A1B LED	9	3 €	27 €	Seoul Viosys	Electronics
Plexisglas – 3 mm	0.1 m^2^	4 €	4 €	Hardware-store	Construction material
Aluminium sheet – 1 mm	0.05 m^2^	10 €	10 €	Hardware-store	Construction material

## 5 Build instructions

The electronic components must be soldered together as indicated in the circuit diagrams. Working with dangerous voltages is not necessary, as the components are operated with laboratory power supplies. The Arduino sketches must then be compiled via the Arduino IDE and flashed to the microcontroller. The LEDs are glued and soldered with thermal glue to the appropriate places on the aluminum plate. Threaded rods can be attached to the outer corners of the aluminum plate to adjust the distance to the catalyst. Alternatively, a device for clamping in a laboratory stand can be used. The reaction vessel is assembled from the plexiglass parts and equipped with appropriate connections for sampling device. In order to be able to insert the catalyst frame into the container at the same height, either plastic screws of the appropriate length can be used to screw the two catalyst frames together, or small Plexiglas rods can be placed in the container on which the catalyst holder rests. The housing for the magnetic stirrer and the sample pump must be cut out accordingly and the components glued or screwed in on the inside. The computer fan, which is used as a magnetic stirrer, is equipped with two neodymium magnets on the axis about 1 cm from the center, which must have opposite polarity at the top. The fan can be screwed into the housing cover from below. The reaction tank is mounted over the pump according to the illustration. The reaction vessel is connected to the flowthrough cuvette via the pump, which is then inserted into the corresponding UV–VIS spectrometer.

## 6 Operation instructions

The system can be used with or without an online UV–VIS spectrometer. The instructions shown here demonstrate the use with online UV–VIS and planar photocatalyst materials from Lynatox GmbH. When operating the light source, care must be taken to ensure sufficient UV protection, as the radiation is intense UV-A and requires protective goggles and a UV protective screen during the measurement. Before the experiment, the photocatalyst samples should be conditioned under the UV source for at least 15 min to remove any residues on the catalyst. In principle, the measurement can be performed with any UV–VIS measurable dye, but there should be no significant absorption bands in the range of the UV wavelength of 365 nm. A possible correction of the photonic calculations for UV adsorbing ingredients is presented in the next section. After conditioning of the catalyst, a measurement of the dye solution without catalyst should be performed to exclude photolytic degradation under UV light. The measurement can be done in 2 modes:

1. Determination of the degradation without differentiated determination of the adsorption rate. Here, the absorbance of the dye solution is determined immediately with the light source switched on and thus the superimposed adsorption is measured with the degradation. This is particularly useful for samples and dyes with low adsorption on the catalyst and simplifies the calculation and determination of the exponential degradation kinetics.

2. Determination of the adsorption rate and subsequent degradation under UV light. Here, the adsorption rate and the degradation rate can be determined separately from each other. This is useful when studying porous catalysts with high adsorption capacity, since adsorption can also stimulate degradation of the dye in solution. Thus, with the setup shown, photolysis, adsorption and degradation can be determined dynamically with a high temporal resolution.

## 7 Validation and characterization

### 7.1 Dyes used and calibration

The organic dyes methylene blue, methyl orange and rhodamine B were used for the exemplary experiments as common substrates for photocatalytic experiments [8]. Since dyes with adsorption bands in the UV-A range would falsify the calculation of the photon efficiency, an absorption spectrum of the 40 µmol/l dye solutions was recorded. The spectrum was recorded in the 200 to 900 nm range using a WTW 7600 UV–VIS, which was also used for the kinetic measurements discussed later. The emission spectrum of the LED light sources was measured in the same wavelength range using an Ocean Optics 650 UV–VIS radio spectrometer with fiber and cosine corrector. The largest overlaps with the excitation wavelength of the LEDs of 365 nm are found for methyl orange and rhodamine B. The measurement wavelength for the actual experiments was determined from the spectra. Furthermore, equidistant calibrations of the dyes in the range of 4 to 40 µmol/l were recorded to determine the limits of detection. The limit of quantitation (LOQ) is three times the limit of detection (LOD). Rhodamine B is most sensitive here. The data for the respective dyes are given in Table 1.

**Table 1:** Photometric calibration data for metylenblue, methylorange and rodamin B.

**Substance**	**Ext/µmol**	**LOD [µmol/l**	**LOQ [µmol/l]**	λ**[nm]**	**R^2^**
**Methyleneblue**	0,0434	0,97	2,91	640	0,999
**Methylorange**	0,0139	0,40	1,20	500	0,999
**Rhodamin B**	0,0475	0,29	0,87	554	0,995

Fig. 2 shows the full spectra of the dyes studied and the LEDs used. All substances show a very low absorption in the UV-A range, so that a photolytic influence on the photocatalytic experiments is not to be expected. Subsequently, the dye concentrations were measured via absorbance at each dyes maximum peak (640, 500, 545 nm). The basic calibration is shown in Fig. 3.

### 7.2 Photonic simulation of light sources

**Fig. 4:**
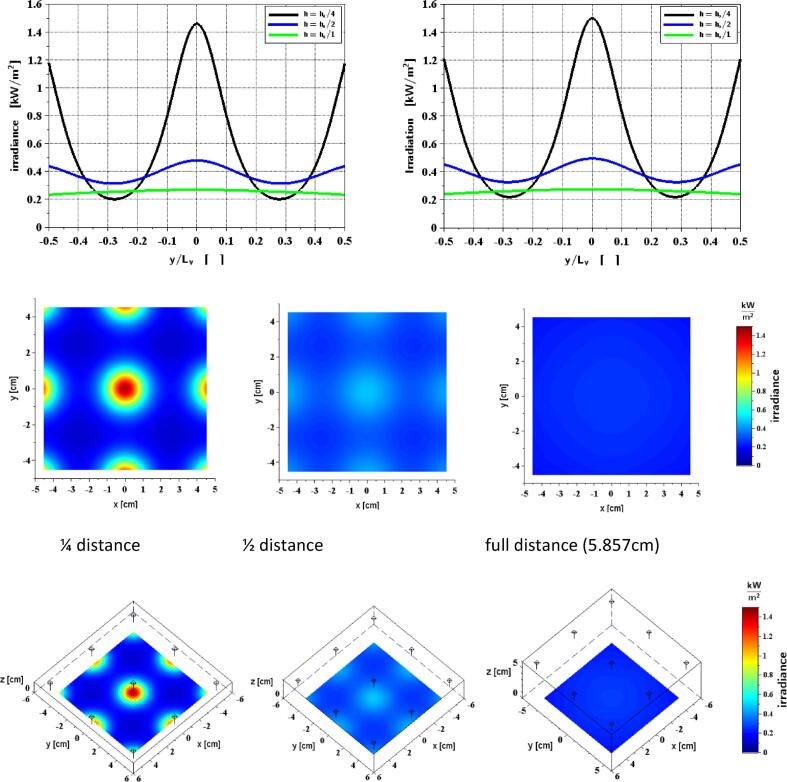
Results of photonic calculations. Top: Irradiance ∂Φe∂A along the midline of the module with (left) and without (right) consideration of reflection losses using Fresnel-equations. Middle: Irradiance as color plot for different LED-spacing. Bottom: The same with representations of LED positions.

To perform a precise calculation of the photon efficiency, it is necessary to project a homogeneous light field onto the 9x9 cm catalyst during the experiments. For this purpose, a photometric simulation of the light source was performed and the incoming light power on the catalyst surface was calculated as a function of the distance between the LEDs and the catalyst. For the LEDs with a beam angle of 120°, each LED produces a beam cone that projects the LED energy onto the catalyst. In good approximation this corresponds to Lambert's cosine law with a constant luminosity L. The inhomogeneity of the light field increases as the distance is reduced, but the irradiance (received light power per area) increases. In the experimental setup, a distance of 58 mm between the LED-plane and the catalyst was selected. At this distance, a homogeneous light field with an integral light power of 2 W is produced on the catalyst surface of 9x9 cm, which corresponds to a irradiance of 246.9 W/m^2^. The radiometric power of the LEDs is linearly dependent on the current of the constant current source, thus the photon current of the light source can be adjusted by the PWM input of the constant current source. The following Fig. 4 shows the luminous field at three distances (5.8 cm, 2.9 cm and 1.45 cm) to the catalyst. The calculation of the wavelength dependent photon current is done according to equation (1) and is needed later for the calculation of the photon efficiency.

(1) $$N_{\mathrm{Photon}}=\frac{\Phi_{e}}{E_{\mathrm{Photon}}}=\frac{\Phi_{e}\lambda }{h_{p}c}$$

Here $\Phi_{e}$ is the radiant flux, λ the wavelength, $h_{p}$ the Plank constant and c the speed of light in vacuum.

For the radiant flux, the following integral over the emitting and receiving surfaces $A_{e}$ and A has to be evaluated numerically:

(2) $$\Phi_{e}=\int_{A}\int_{\mathrm{Ae}}L\frac{\cos\left(\beta \right)\cos\left(\beta^{\prime }\right)f_{\mathrm{Fresnel}}\left(\beta \right)}{{\left|{\vec{r}}_{e}-\vec{r}\right|}^{2}}dA_{e}dA$$

where

(3) $$\cos\left(\beta \prime \right)=\frac{{\vec{r}-\vec{r}}_{e}}{\left|{\vec{r}}_{e}-\vec{r}\right|}∙\frac{d{\vec{A}}_{e}}{dA_{e}}$$

and

(4) $$\cos\left(\beta \right)=\frac{{\vec{r}}_{e}-\vec{r}}{\left|{\vec{r}}_{e}-\vec{r}\right|}∙\frac{d\vec{A}}{\mathrm{dA}}$$

are the directional cosines between the ray emitted from surface element $d{\vec{A}}_{e}$ at location ${\vec{r}}_{e}$ and received by surface element $d\vec{A}$ at location $\vec{r}$. Finally, $f_{\mathrm{Fresnel}}\left(\beta \right)$ is the fraction of power transmitted through the liquid surface. For this purpose, we use in this work the well-known Fresnel formulas with an isotropic refractive index of n = 1.34 for water and n = 1.0 for air. Equation (2) is implemented here with the scripting tool SCILAB® in such a way that the surfaces are triangulated into small areas. Thus, the integrals can be performed easily and very accurately with Gaussian quadrature. Moreover, this method allows the treatment of curved surfaces, e.g. by specifying parametrized surface patches. From Fig. 4 it can be concluded that the reflection of the incident power at the water surface is negligible in the present case.

### 7.3 Determination of photolysis, adsorption and degradation rate and photon efficiency

**Fig. 5:**
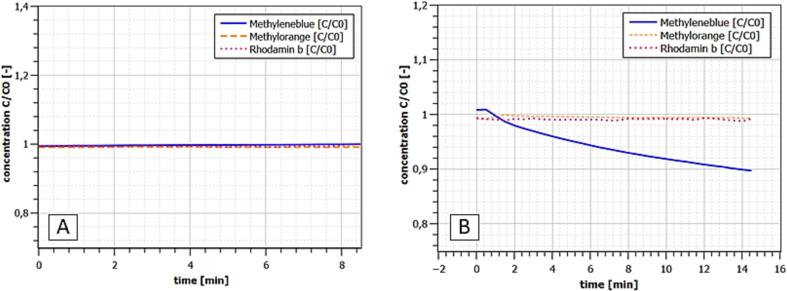
Measured dye concentrations for UV-A photolysis (A) and adsorption (B); one experiment for each dye.

For exemplary validation of the presented experimental set-up, experiments on the photocatalytic degradation of the dyes methylene blue, methyl orange and rhodamine B were carried out. All dyes were fed to the reactor at initial concentrations of 40 µmol/l. For each dye, one experiment was performed for UV-A photolysis, adsorption on the catalyst, degradation after adsorption phase and degradation without upstream adsorption phase. The purpose is to show the functionality of the experimental set-up and to give recommendations for performing and evaluating photocatalytic degradation experiments. The catalyst used consists of a fiberglass grid (mesh size 0.5 mm), which was coated with a suspension of anatase-modified titanium dioxide nanoparticles of 14 nm diameter. The anatase content of the titanium dioxide was 90 % and the Brunauer–Emmett–Teller (BET) surface area of the particles was 50 m^2^/g. The measured concentration profiles for photolysis and adsorption are shown in Fig. 5.

No photolytic degradation was observed for any of the dyes in ultrapure water solution. Only methylene blue showed relevant adsorption on the catalyst. A 10 % decrease in concentration was measured even after 14 min, with the onset of saturation being evident. For all dyes an exponential photocatalytic degradation behavior was shown, whereby the substance-specific degradation rates can be ordered as follows: Methylene degradation > Methyl orange > Rhodamine B. The exponential degradation behavior found can be explained by the Langmuir-Hinshelwood (L-H) model [11], [12]. Here, the reaction rate can be calculated as follows:

(5) $$r=-\frac{\mathrm{dC}}{\mathrm{dt}}=k_{\mathrm{LH}}\frac{K_{L}C_{\mathrm{eq}}}{1+K_{L}C_{\mathrm{eq}}+\sum_{i=1}^{n}K_{i}C_{i}\left(i=1,n\right)}$$

Where r is the reaction rate, $k_{\mathrm{LH}}$ is the specific rate constant, $K_{L}$ is the Langmuir constant, $C_{\mathrm{eq}}$ is the liquid concentration. $K_{i}$ and $C_{i}$ are the Langmuir constant and concentration of each substance within the solution. For small reactant concentrations and absence of other dissolved compounds, a change in the term $\frac{K_{L}C_{\mathrm{eq}}}{1+K_{L}C_{\mathrm{eq}}+\sum_{i=1}^{n}K_{i}C_{i}\left(i=1,n\right)}$ is not expected during the reaction period, so a simplification to first-order kinetics is possible:

(6) $$r=-\frac{\mathrm{dC}}{\mathrm{dt}}=kC_{\mathrm{eq}}$$

(7) $$\mathrm{HT}=\frac{\ln(2)}{k}$$

**Fig. 6:**
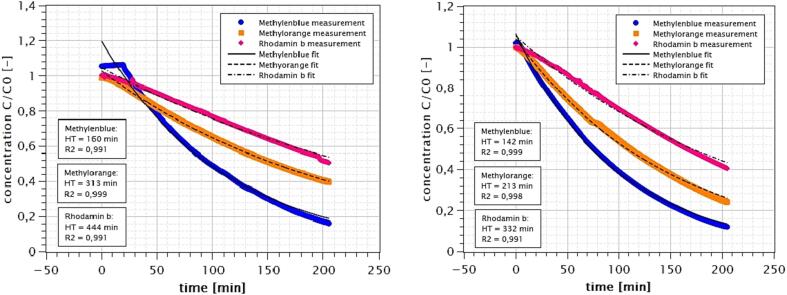
Concentration profiles during photocatalytic treatment without (left) and with (right) separate adsorption phase; one experiment for each dye.

For improved readability, calculated 1st order constants were converted to half-time values (HT) according to formula 7. The concentration profiles of the photocatalytic oxidation with and without prior adsorption phase are shown in Fig. 6. For methylene blue, the only substance with significant adsorption affinity, a concentration plateau is visible at the beginning of the experiment without upstream adsorption. Later on, both tests show similar degradation behavior.

**Fig. 7:**
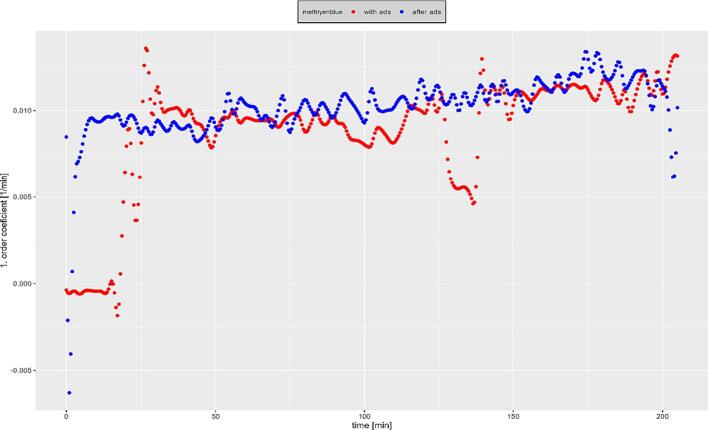
First order constants for methylenblue photocatalytic degradation with and without adsorptionphase.

To further investigate the influence of this prior adsorption phase on measurable reaction rates, a spline interpolation of the measured values was carried out with subsequent calculation of the measured reaction rates and first order constants over time. The results for methylene blue are shown as an example in Fig. 7. Here, the influence of the prior adsorption phase is clearly evident in the first 30 min of the reaction period. Subsequently, identical 1st order constants are achieved within the measurement accuracy, so that a simpler test procedure without an upstream adsorption phase is possible without loss of information. Similarly, the preceding adsorption phase can be replaced by calculating the difference between individual adsorption and degradation curves.

Consequently, the dyes studied differ in their adsorption and degradation affinities. The observed adsorption affinity of methylene blue is attributable to its charge opposite to that of the surface of the catalyst, following the knowledge gained on point of zero charge (POZC) of photocatalysts. Following this, an investigation using an adsorbing dye (methylene blue) and a non-adsorbing dye (methylorange) is recommended, so that the reactivity with adsorbed (hydroxyl) radicals as well as those diffused into the boundary layer can be assessed. The low adsorption of methyl orange and rhodamine B also makes it possible to simplify experiments without or with a very short initial adsorption phase. Based on these reaction rates and the quantum flux determined according to formular 1, the photon efficiency was also calculated over the duration of the experiments. Based on the extinction measurements of the individual dyes an corrected photon efficiency was calculated according to following formular, whereby the losses via radiation absorption within the fluid are considered:

(8) $$n=\frac{r_{i}*N_{A}}{N_{\mathrm{Photon}}{*10}^{-\varepsilon *c*d}}$$

**Fig. 8:**
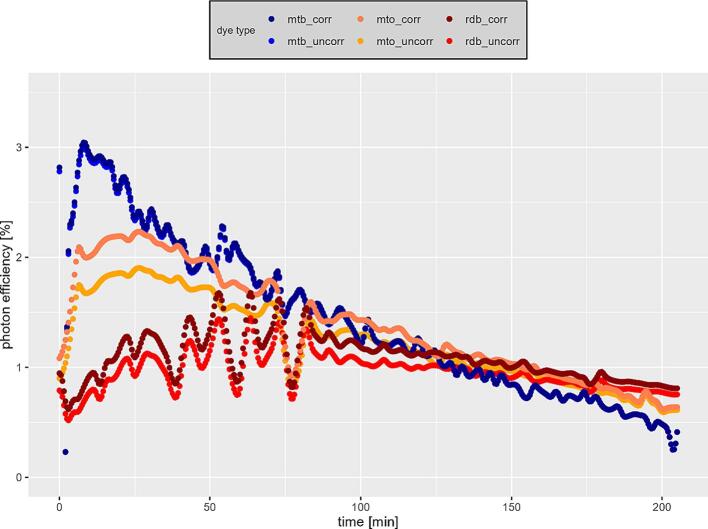
Quantum efficiency for photocatalytic dye degradation over reaction period.

Here $r_{i}$ is the reaction rate of a dye at the respective time, $N_{A}$ is the Avogadro constant, ε∗c is the product of the extinction coefficient (365 nm) and the respective substance concentration, and d is the layer thickness of the water over the catalyst. The results for all dyes are shown in Fig. 8. Quantum efficiencies of 0.5–3 % were achieved over the reaction time, clearly showing the influence of first-order kinetics. Following the measured extinction spectra, a moderate influence of the radiation absorption on measured photon efficiencies is shown for methyl orange and rhodamine B.

Compared to literature values in [10], [13], the high photon efficiencies in the first 50 min of the reaction are noteworthy. One explanation for this is the higher concentration of the initial solutions (40 µmol/l). [13] achieved efficiencies of 0.02–––0.03 % with a 10 µmol/l methlyene blue solution (Din 52,980 experimental set-up) with plasma sparyed TiO2 coatings. Likewise, [14] using Degussa P25 nanoparticles under an initial methylene blue concentration of about 65 µmol/l achieved photon efficiencies of 0.3–––2.4 %. The irradiance of approx. 250 W/m^2^ used was comparatively high (10 W/m^2^ according to DIN 52980), so that an increase in photon efficiency can be expected for lower values.

In conclusion, the presented experimental setup provides a low-cost and modular possibility to perform photocatalytic experiments. The modular design allows easy adaptation of reactor, online measurements and light sources. The presented optical calculation can be transferred to monochromatic LEDs of variable wavelength, provided that an identical radiation pattern is present (radiation angle 120°). In addition to the use of a UV–VIS measurement, the setup also allows an additive integration of further flow measuring cells (pH, oxygen, etc). Adaptation of the reactor shape to specific shapes of planar catalysts can be implemented cost-effectively thanks to the plexiglass design. This enables designs with low hydraulic volumes, which can significantly reduce the duration of experiments.

### CRediT authorship contribution statement

**Tobias Schnabel:** Conceptualization, Methodology. **Robert Honke:** Visualization. **Andreas Schmid:** . **Simon Mehling:** Visualization. **Rene' Göhring:** Visualization. **Oldrich Simek:** Data curation. **Axel Wolfram:** Data curation. **Andre Wetterauer:** Visualization. **Christian Springer:** .

## Declaration of Competing Interest

The authors declare that they have no known competing financial interests or personal relationships that could have appeared to influence the work reported in this paper.
